# News and Coming Events

**Published:** 1907-09-21

**Authors:** 


### NEWS AND COMING EVENTS.

The annual dinner of the St. George's Hospital will take place at the Whitehall Rooms, Hôtel Metropole, at 7 p.m. on October 1 next.

Sir Samuel Wilks, F.R.S., will take the chair at the opening meeting of the Guy's Hospital Pupils' Physical Society on October 4 next, at 8 p.m., when Dr. G. A. Gibson, of Edinburgh, will read a paper entitled "Past and Present."

On October 2 next Professor Osler, F.R.S., Regius Professor of Medicine at Oxford, will deliver the introductory address at St. Mary's Hospital Medical School.

"The present state of medical education in London" will be the theme of Dr. W. H. Allchin's address at the opening of the Westminster Hospital Medical School on October 1 next.

The annual festival service of the Guild of St. Luke will be held in St. Paul's Cathedral on October 22. Medical practitioners who desire to attend are requested to communicate with the Registrar, Mr. Claude St. Aubyn-Farrer, Westbourne Park Road, W.

The Committee of the South Devon and East Cornwall Hospital, Plymouth, has received a summons—which will be heard at the Guildhall, Plymouth, at eleven o'clock on Monday morning next, September 23—for the payment of taxes on men-servants in uniform. The case will be one of great interest to all hospitals.

The Second Norman Kerr Lecture, under the auspices of the Society for the Study of Inebriety, will be delivered by Dr. Robert Welsh Branthwaite (H.M. Inspector under the Inebriates Acts) on Tuesday, October 8, 1907, at 8.30 p.m., in the Hall of the Royal Society of Medicine, 20 Hanover Square, London, W. Subject: "Inebriety; its Causation and Control."

To meet the ever-increasing demand and opening for temperance work the Women's Total Abstinence Union is organising a National Bazaar, to be held in the Caxton Hall, Westminster, on December 10, 11 and 12, by which to raise £5,000 for this purpose. Contributions will be gratefully received by the treasurer, Mrs. E. W. Brooks, Duvals, Grays, Essex, or by the hon. secretaries, Miss Hilda Dillon, 47, Oakley Street, Chelsea, and Miss Florence Brooks, Duvals, Grays, Essex.

At the annual meeting of the Governors of the Salisbury Infirmary attention was drawn to the numerous improvements which had been effected during the year. The operation theatre has been remodelled and is now thoroughly up-to-date, and a new *x*-ray apparatus has been provided. The lift in the central hall, opened in November last by H.R.H. Princess Christian, has proved of great use, and an immense boon to patients and staff. The costs of these various improvements have been met by friends, and have in no case been charged to the general accounts of the institution.

Dr. William Ewart will deliver the inaugural address of the winter session at St. George's Hospital Medical School on Tuesday, October 1 next. His address will be entitled "Res medica, res publica."

The Waltham Joint Hospital Board has decided to increase the accommodation of the Waltham Joint Hospital at an estimated cost of £4,100. The plans provide for the erection of a new 16-bed block and additions to the administrative block.

Stockton and Thornaby Hospital has had its accommodation considerably increased by the completion of the new extensions. The new buildings cost £12,000, of which nearly £10,000 has been contributed by local firms and friends of the institution. A special workmen's fund has been raised to support the extension building fund, and already amounts to nearly £2,000.

The inaugural address of the winter session of the London School of Clinical Medicine, Seamen's Hospital (*Dreadnought*), Greenwich, S.E., will be delivered in one of the lecture rooms of the School by Sir Richard Douglas Powell, Bart., K.C.V.O., M.D. (President, Royal College of Physicians), on Tuesday, October 8, 1907. The chair will be taken at 3.30 p.m. by Perceval A. Nairne, Esq. (Chairman of the Board of Governors). The school and hospital will be open for inspection. Tea will be served after the address.

The opening lecture of the autumn course of post-graduate classes in connection with the Glasgow Royal Infirmary was delivered in the Dispensary Hall on the afternoon of the 3rd inst. by Sir Alemroth Wright, M.D., F.R.S., whose subject was "Vaccine Therapy." In the course of his remarks the lecturer said he was thoroughly in sympathy with the graduate study movement. The teaching medical men got as students was practically nothing, and, when every year medical science was advancing, it was absolutely necessary to keep abreast of new discoveries. He was of opinion that vaccine therapy would form an important branch of study in the medical schools of the future.

An Island Without a Public House.—Mr. F. N. Charrington, who has devoted his life to efforts for the reclamation of inebriates, has arranged to receive gentlemen (the victims of alcoholic excess) as "paying guests" on Osea (pronounced "O.C.") Island, which he purchased in 1903. The island, which is in Blackwater Bay, Essex, is reached by the twin screw steamer "Annie," running twice daily from Maldon East, five miles distant. Residents are absolutely removed from temptation, as no alcohol of any description can be obtained in the whole of the territory. In many homes for inebriates the inmates are confined within four walls, on the other side of which public houses, with all their temptations, are frequently near at hand. On Osea Island, however, there is full liberty within its four-mile radius. The needful recreation is provided by ideal golf links, and there is every facility for sea bathing and sea fishing. The home is intended for the reception of gentlemen only of the upper and upper middle classes. Those interested in the movement can obtain an illustrated guide and full particulars by writing to Mr. F. N. Charrington, Osea Island, Heybridge, Essex.

